# Detecting neurobiological markers in treatment response to prolonged exposure therapy for PTSD: An RCT using functional near-infrared spectroscopy

**DOI:** 10.1016/j.mex.2026.103867

**Published:** 2026-03-19

**Authors:** Duane D. Booysen, Sizwe Zondo

**Affiliations:** Rhodes University, Department of Psychology, Faculty of Humanities, 1 University Road, Makhanda, 6139, South Africa

**Keywords:** Functional near-infrared spectroscopy, Prolonged exposure therapy, PTSD, Randomized clinical trial

## Abstract

Trauma exposure and post-traumatic stress disorder (PTSD) are a global public health concern, especially in low and middle-income countries (LMICs). University students are a unique population that has been found to have increased levels of trauma exposure and PTSD, yet limited clinical outcome and neurobiological data exist, especially in LMICs, on treatment responses to evidence-based trauma-focused treatments for PTSD among university students in LMICs. The development and integration of neuroimaging tools in psychotherapy enable more robust integration of data from clinical psychology and neuroscience. Our protocol is the first to use functional Near-infrared spectroscopy (fNIRS) to investigate the treatment response of a first-line trauma-focused cognitive behavioural therapy, namely, prolonged exposure therapy, for the treatment of PTSD. Using a pilot randomised controlled trial, university students diagnosed with PTSD will be assessed using clinical outcome measures and fNIRS to ascertain prefrontal cortical increases and decreases in persons diagnosed with PTSD. Participants will be randomised to either the experimental condition (PE) or a comparative control condition (supportive counselling). Participants will be assessed at baseline, post-intervention, and at 12 and 24-week follow-ups using the same measures and imaging.

• Recruit and assess university students using standard clinical measures and fNIRS for PTSD.
• Assign enrolled students to either PE (experimental condition) or supportive counselling (control condition) for a minimum of 8 sessions.
• Assess students at the end of treatment and at 12 and 24-week follow-ups using the same clinical measures and fNIRS for any changes in PTSD.

## Graphical abstract

**Figure:**
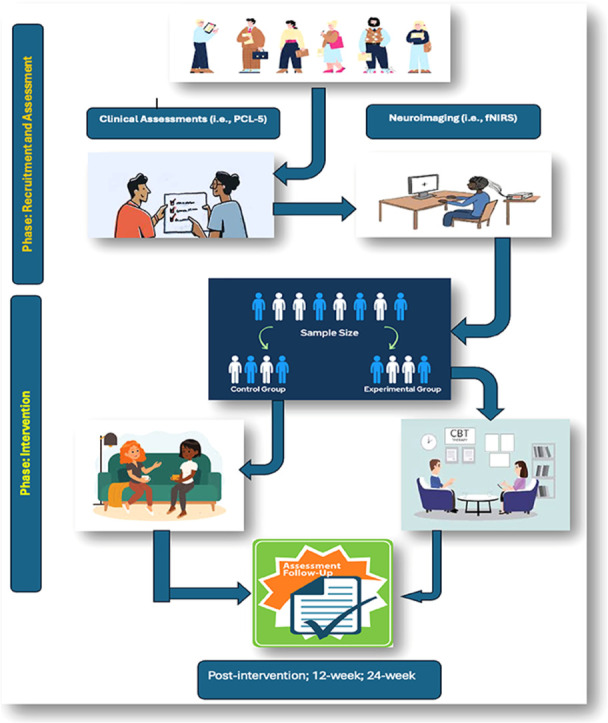

Specifications table

**Table:** 

**Subject area**	Psychology
**More specific subject area**	Functional Near-infrared Spectroscopy and Prolonged exposure therapy for PTSD
**Name of your protocol**	Detecting Neurobiological Markers in Treatment Response to Prolonged Exposure Therapy for PTSD: An RCT using Functional Near-infrared Spectroscopy
**Reagents/tools**	PTSD Checklist for DSM-5 (PCL-5) Patient Health Questionnaire - 9 (PHQ-9) The Generalised Anxiety Disorder Questionnaire seven item (GAD-7) Alcohol Use Disorder Test (AUDIT) Functional Near-Infrared Spectrometry (NIRxSport2, NIRx Medical Technologies, LLC, Berlin, Germany)
**Experimental design**	This is a two-armed comparative pilot randomized controlled trial*.*
**Trial registration**	Not Applicable.
**Ethics**	This study was approved by the Human-Research Ethics Committee of Rhodes University (Reference number: 2025–8317–9503). Participants will sign the Informed Consent Form online and data confidentiality will be guaranteed.
**Value of the Protocol**	First randomized controlled trial to evaluate the effectiveness of an abbreviated version of prolonged exposure therapy for PTSD in South Africa. Frist RCT to assess the effectiveness of fNIRS to detect a treatment response for PTSD in a South African sample. First RCT to evaluate the feasibility of fNIRS as a routine neuro-imaging measure in psychotherapeutic clinical practice.

## Background

A national student mental health survey (*n* = 28,268) across 17 universities in South Africa found, among others, that PTSD (21%) was among the highest disorders among students [1]. Compared to earlier epidemiological research on common mental disorders in South Africa, the prevalence of PTSD (21.0%) among university students is higher than among the country's general population [2] and suggests that students may be exposed to high levels of trauma, as found in previous SA studies [3].

Several evidence-based trauma-focused psychotherapies have been developed for PTSD [4]. Prolonged exposure therapy (PE; [5]) has accrued substantial evidence for demonstrating its efficacy and effectiveness in treating PTSD and comorbid disorders such as depression, anxiety, and alcohol use [6,5]. An abbreviated version of PE, PE for Primary Care (PE-PC; Rauch et al., 2023), has been developed to increase accessibility to effective trauma-focused treatments for PTSD. Yet limited research exists on the efficacy of PE-PC in low- and middle-income countries (LMICs) such as South Africa. Notwithstanding the progress in developing effective treatments for PTSD, the measurement of treatment response in psychotherapy is limited due to its sole reliance on structured clinical interviews and self-report measures [7,8]. The use of neuroimaging to determine neurobiological markers has received increased attention to support the validity of treatment outcomes in ameliorating PTSD [9]

Biological models of PTSD, particularly as indicated by functional magnetic resonance imaging (fMRI) and subsequent trauma exposure, suggest increased activation in the dorsolateral prefrontal cortex (dlPFC) and ventrolateral PFC (vlPFC) in response to negative emotion stimuli [10,11]. Yet the utility of fMRI in routine clinical practice remains costly and impractical, especially in LMICs such as South Africa. Moreover, neuroimaging research is mainly focused on high-income countries, which limits the generalizability of the current literature base; therefore, integrating neuroimaging techniques and investigating neurobiological outcomes are imperative [12].

Functional near-infrared spectroscopy (fNIRS) neuroimaging uses light in the near-infrared (IN) spectrum range (650–950 nanometres) (red light) to detect the concentration of oxygenated haemoglobin (HbO) and deoxygenated haemoglobin (deoxy-Hb), activated within the cortex in response to neuronal activation [13]. As a biosensor, fNIRS measures the interaction between light and matter and how much of the light is absorbed by haemoglobin - a metalloprotein that carries oxygen from the alveolus to the rest of the body via red blood cells. fNIRS neuroimaging, therefore, seeks to gather brain activity by measuring changes in hemodynamic responses, as indicated by changes in the concentration of haemoglobin protein in neuronal cells [14]. Moreover, fNIRS is a low-cost, radiation-free and portable neuroimaging instrument indicated for clinical-based evidence-based psychotherapy [15]. To this end, fNIRS combined with standard psychological assessments provides an opportunity for more accurate assessment and measurement of treatment-response-based brain activity in persons diagnosed with PTSD.

Taken together, the study aims to (1) compare the effectiveness of PE-PC to standard care, such as supportive counselling (SC), for treating PTSD among students, corroborated by fNIRS. Secondarily, the study aims to (2) ascertain the effectiveness and feasibility of fNIRS to detect treatment responses, to increase the validity of psychotherapy outcomes.

## Description of protocol

### Method and design

#### Ethical approval

The study received ethical approval from the Rhodes University Ethics Committee [2025–8317–9503]. A pilot comparative randomised controlled trial, comparing PE-PC to Supportive Counselling (SC) will be used with fNIRS as a neurobiological measurement of treatment-response. The present study has four broad aims, namely, (1) to investigate the efficacy of fNIRS optical neuroimaging to detect decrease in the dorsolateral prefrontal cortex (dlPFC) and ventrolateral prefrontal cortex (vlPFC) involvement in PE-PC for PTSD. Second, (2) whether online delivered PE-PC can result in greater outcomes compared to supportive counselling among university students diagnosed with PTSD as the primary outcome. Then, (3) whether PE-PC providers and clients experience and perceive PE-PC as a feasible and acceptable treatment for PTSD. Lastly, (4) how clients experience and perceive fNIRS as a neuroimaging tool as part of psychotherapy assessments for PTSD. The following research hypothesis and questions will guide the proposed study:

***Behavioural Treatment Outcomes:***

• Hypothesis 1: PE-PC will result in larger reductions in PTSD severity [Outcomes: (PCL-5; Week 0 to Week 6) compared to SC; these differences will be maintained at 12 weeks and 24 weeks post-treatment.
• Hypothesis 2: PE-PC will result in larger improved symptoms of depression (PHQ-9; Week 0 to Week 6), compared to SC, and these differences will be maintained at 12 weeks and 24 weeks post-treatment.
• Hypothesis 3:PE-PC will result in larger reductions in alcohol use (AUDIT) (Week 0 to Week 6) compared to SC, and these differences will be maintained at 12 weeks and 24 weeks post-treatment.
• Hypothesis 4: PE-PC will result in larger improved symptoms of anxiety (GAD-7; Week 0 to Week 6), compared to SC, and these differences will be maintained at 12 weeks and 24 weeks post-treatment.

***Neurobiological outcomes:***

• Hypothesis 5: fNIRS will accurately detect a decrease dlPFC and vlPFC among persons who completed a minimum of 4–8 sessions of PE-PC for PTSD.
• Hypothesis 6: PE-PC will result in a greater decrease in dlPFC and vlPFC compared to SC among persons who completed a minimum of 4–8 sessions of PE-PC or SC for PTSD.

***Implementation outcomes:***

• How do treatment providers experience and deliver PE-PC online for persons diagnosed with PTSD?
• How do persons diagnosed with PTSD experience receiving PE-PC online?
• How do persons diagnosed with PTSD perceive and experience fNIRS as a neurobiological imaging instrument to assess treatment response?

### Research setting

A public university will be the general setting of the study, and a student population sample will be recruited to participate. The university has internet and technology infrastructure to support the delivery of online psychological interventions to students.

### Participants

The study will be inclusive of persons older than 18 years of age who are registered students at Rhodes University. Persons reported to have directly experienced or witnessed a traumatic event(s) and who report significant symptoms of PTSD and dysfunction, as measured by the PTSD Checklist-5 (PCL5 ≥ 28) will be included. Significantly, participants should have experienced PTSD and dysfunction for at least 3 months post-trauma. Participants who meet the minimal inclusion criteria will be randomly assigned to either the PE-PC or SC group. The proposed sample size was determined a priori to be *N* = 34, per group, for a power = 0.8; to detect an effect greater than *d* = 0.71. Sample estimation was calculated and based on a previous RCT on PE-PC [16].

### Treatment providers

The providers will be Registered Counsellors (RCs), hereafter referred to as providers, who will provide either PE-PC or SC remotely. The interventions will be provided via online platforms such as Zoom, Google Meets, and MS Teams. RCs are registered with the Health Professions Council of South Africa (HPCSA) as licensed mental health practitioners to provide psychological services to the public. The delivery of PE-PC and SC will be done online. Therefore, providers will be based in various locations in South Africa. Yet clients will all be based and recruited in Makhanda and on the Rhodes University Campus. The research team consists mainly of a clinical psychologist (DB) and a neuropsychologist (SZ) and a graduate student completing her PhD.

### Intervention

#### Experimental condition: prolonged exposure therapy for primary care (PE-PC)

PE-PC is an abbreviated version of the standard PE model for the treatment of PTSD (See Appendix A). PE-PC has a minimum of 4–8 weekly sessions, each lasting 30 min, that consist of imaginal exposure and emotional processing in a written format, and in vivo exposure activities between contact sessions. During contact, providers conduct an assessment, provide psychoeducation and explain the rationale of PE-PC. In contacts 1–4, participants complete a written exercise on trauma memory, engage in processing questions related to the trauma, and engage in vivo activities. At each session, participants will read the written account or the memory and answer the processing questions. Providers are tasked with processing responses during the contact and providing encouragement and support where necessary. As in standard PE, the goal is for an individual with PTSD to experience and learn that the fear/distress associated with traumatic memories can be reduced through exposure to reminders of the trauma and the trauma memories.

#### Control condition: supportive counselling

Supportive counselling (See Appendix B) will be the controlled condition used as it aligns with standard mental healthcare in South Africa. Participants assigned to this condition will receive 4–8 sessions of 30 min of SC, which consists of non-directive person-centred principles such as empathy, non-judgemental stance, congruence, and the notion that persons will.

### Neurobiological assessment: functional near infrared spectrometry (fNIRS)

**Fig. 1:**
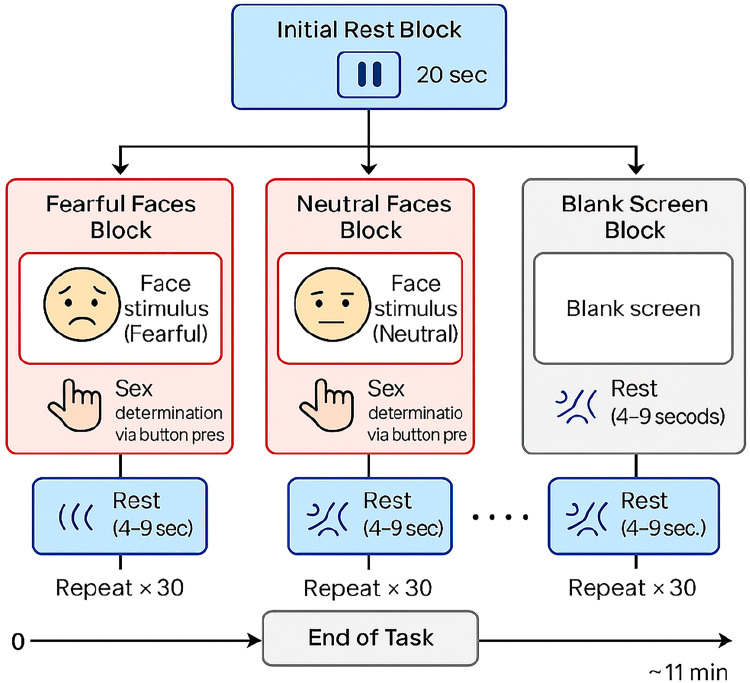
The facial expression paradigm.

A rapid response paradigm will be used to activate brain activity in participants diagnosed with PTSD (See [7,17]). The study’s rapid facial expression paradigm will be based on Balters et al. [7], who found significant cortical activation in response to facial stimuli, with increased oxygenated haemoglobin activation in the left dlPFC for fearful faces and increased bilateral activation in the dlPFC for neutral faces. Similar to their study, our fNIRS block design will comprise a total of thirty fearful (F) faces, thirty neutral faces (N), and thirty blank-screen (BS) presented in randomized order. Each block, stimuli (F or N) or BS will be presented for 2 s, followed by a blank screen, lasting between 4 and 9 s to ensure jittered inter-trial-intervals. Significantly, interspaced rest periods at the beginning and end of experiment will be incorporated into the paradigm as depicted in Fig. 1. All event markers (triggers) will be programmed on PsychoPy and sent to the Aurora Acquisition Software (NIRx, Medical Technologies, LLC, Berlin, Germany), via lab streaming layer (LSL).

### Functional near-infrared spectrometry

#### Data acquisition and montage

**Fig. 2:**
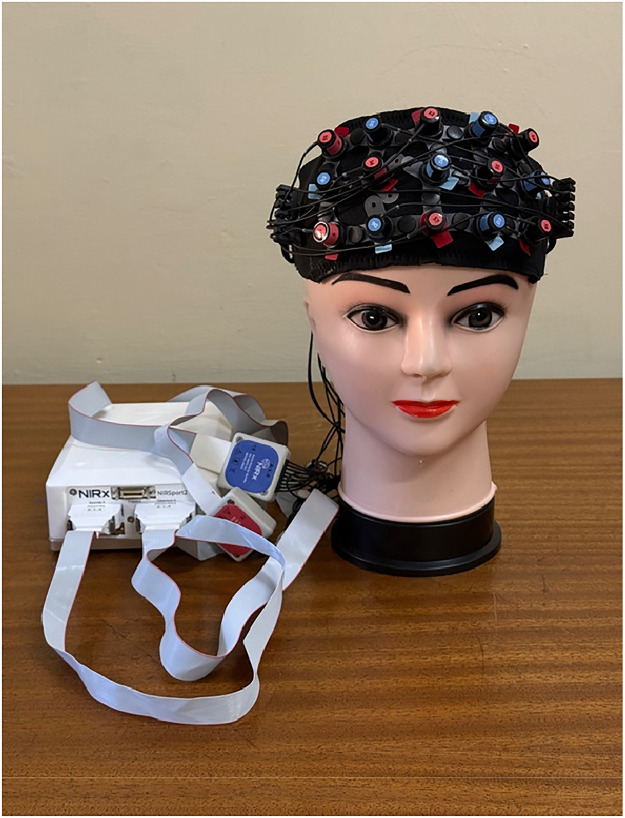
fNIRS Headband (EasyCap, NIRx, medical technologies, LLC, Berlin, Germany).

The measure of cerebral activity based on concentration changes in oxygenated (HbO) and deoxygenated haemoglobin (deOxy). Data will be collected using the NIRxSport2 (NIRx, Medical Technologies, LLC, Berlin, Germany), a portable continuous wave fNIRS device, while participants completed the facial paradigm. For the study, eight LED emitters (sources), will be paired with seven photodiode detectors, covering the prefrontal cortex as indicated in Fig. 2. Optodes will be placed according to the 10–20 system, using a standardized prefrontal fNIRS Headband (EasyCap, NIRx, Medical Technologies, LLC, Berlin, Germany).

**Fig. 3:**
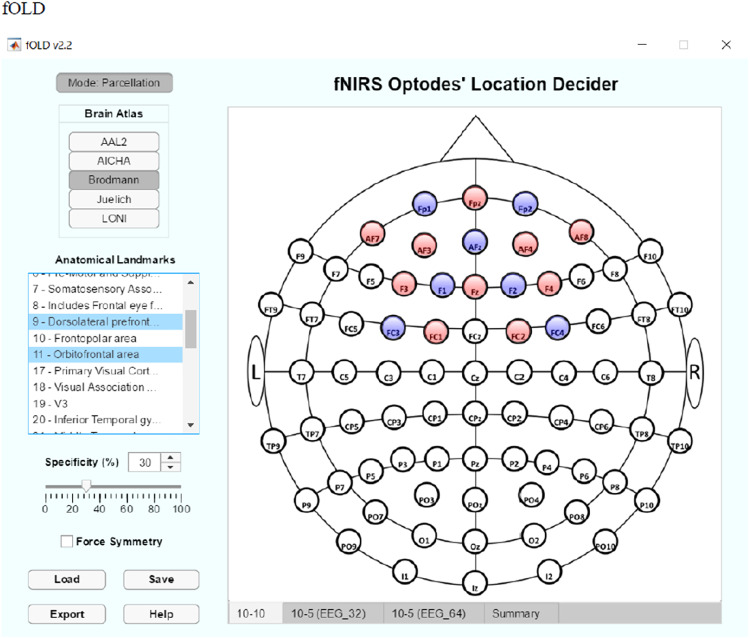
Screen shot of the fOLD software. The software to enable optode location for regions of interest implicated in PTSD (DLPF, Orbitofrontal).

Importantly, probe placement (sources and detectors) to identify the most sensitive placement for each optode location will be determined using the ‘fNIRS Optodes Location Decider’ (fOLD) software (Zimeo [18]) (Fig. 3). Optode locations will be paired with relevant Montreal Neurological Institute (MNI), coordinates using the Aurora Acquisition Software. The placement of sources and detectors will correspond with cortical regions implicated in PTSD, inclusive of the ventrolateral prefrontal cortex, and dorsolateral prefrontal cortices [7]. In total, signals will capture twenty-two channels covering the prefrontal cortex.

#### Provider training and fidelity

The training will consist of two separate workshops, namely, standard prolonged exposure therapy (PE) (2 days) and prolonged exposure for primary care (PE-PC) (2 days), that will run over 4 days, at approximately 7 h per day. DB will lead the PE training to provide a foundation and the rationale of PE. The second workshop will focus on PE-PC and will be led by DB. A registered clinical psychologist will also train providers in SC for 4-days. The study will include the maximum number of eligible providers in the training to offset attrition rates among providers. Post-training consultations will be scheduled during the implementation of PE-PC and SC. DB will hold separate consultations for PE-PC and SC, and consultations will occur weekly during recruitment and intervention.

#### Treatment fidelity

Treatment fidelity is integral to reliable and credible intervention research. Due to all sessions being online, all treatment sessions will be video and audio-recorded to ensure an accurate review of all intervention sessions. Treatment fidelity ensures construct validity in the study; for example, the stated treatment was truly and adequately administered. The fidelity self-report measure will also be used in supervision to discuss any adverse events, concerns, or problems providers may encounter during sessions. A minimum of 20% of recorded sessions will be reviewed by an independent reviewer trained in PE-PC and SC to assess the fidelity of sessions.

### Randomization and masking

All eligible persons, with the assistance of the project statistician, will be randomized to either (1:1) the experimental condition (PE-PC) or the control condition (SC). The consultant statistician will administer randomization. Participants will be blind (single) to who is receiving which treatment.

### Data collection

The proposed study will have 5 time points, as expanded below: (T1) pre-intervention, (T2) during intervention, (T3) post-intervention, (T4) 12-week follow-up, and (T5) 24-week follow-up.

Several assessment measures will be used to collect data. All measures, except the neurobiological measure (fNIRS), will be self-reported. Similar RCTs have used self-report measures to determine PE-PC's effectiveness (see Rauch et al., 2023). The following assessment measures will be used:

**Time 1 - Pre-intervention**

The study participants will be notified about the study via a university email list and posters with a QR code. A URL link will provide interested participants with information about the study, the opportunity to provide consent, basic demographic data, and complete clinical measures that will determine eligibility. The initial measures to be completed by interested persons using the Google Form link will be the PCL-5, PHQ-9, AGD-7, and AUDIT. Once completed, eligible clients will be informed about the date and time to complete the fNIRS paradigm assessment. The paradigm will be completed in person and facilitated by SZ and DB.

### Primary outcomes

• The *PTSD checklist for DSM-5* (PCL-5; [19]) is a 20-item self-report measure that assesses the 20 DSM-5 symptoms of PTSD. The PCL-5 has a variety of purposes, including (a) monitoring symptom change during and after treatment, (b) screening individuals for PTSD, and (c) making a provisional PTSD diagnosis. The PCL-5 test scores demonstrate good internal consistency (α = 0.96), test-retest reliability (*r* = 0.84), and convergent and discriminant validity (Bovin et al., 2015).
• *Functional Near-Infrared Spectroscopy* (fNIRS). fNIRS neuroimaging seeks to gather brain activity by investigating changes in oxygenated (HbO) and deoxygenated (deOxy) hemodynamic, in response to cognitive or behavioural stimuli [14].

### Secondary outcomes

• *Patient Health Questionnaire - 9* (PHQ-9): The PHQ is a 9-item self-administered questionnaire that is used to detect depression severity. In addition to making criteria-based diagnoses of depressive disorders, the PHQ-9 is also a reliable and valid measure of depression severity [20].
• The *Generalised Anxiety Disorder Questionnaire seven item* (GAD-7) [21]. The GAD-7 is a 7-item self-administered questionnaire that is used to detect anxiety related distress. The GAD-7 is a valid and efficient tool for screening for GAD and assessing its severity in clinical practice and research [21].
• The *Alcohol Use Disorder Identification Test* (AUDIT) AUDIT provides a simple method of early detection of hazardous and harmful alcohol use in primary health care settings and is the first instrument of its type to be derived based on a cross‐national study [22]. The AUDIT is a 10‐item questionnaire which covers the domains of alcohol consumption, drinking behaviour, and alcohol‐related problems.

**Time 2 - During Intervention**

Clients will complete the PCL-5 and (PHQ-9) before every session to measure symptomology during treatment. The PCL-5 and PHQ-9 will either be self-completed by the client or with the assistants of the treatment provider. The intervention will consist of PE-PC and SC, and participants will be randomized to either condition.

**Time 3 – Post-Intervention**

Assessments completed in the pre-intervention will be completed at post-intervention, namely, PCL-5, PHQ-9, GAD-7, AUDIT, and fNIRS. Additionally, *Perceived characteristics of Intervention Screen* (PCT; [23]) will also be administered to obtain data on how participants experienced and perceived PE-PC. The PCIS is a 20-item scale on how treatment providers experience and perceive the implementation of an empirically supported trauma-focused treatment for PTSD.

**Time 4 – 12-week follow-up**

Twelve weeks after the post-intervention, the measures used in T1 will be used to assess whether treatment gains have been maintained, improved, or deteriorated.

**Time 5 – 24-week follow-up**

Twenty-four weeks after the post-intervention assessment, the same measures used in the T1 will be used to assess whether treatment gains have been maintained, improved, or deteriorated.

### Data analysis

#### Intervention effects

Intention-to-treat analysis will be used for the study analysis. The method analyses study results in a prospective randomized study where all randomised participants are included in the statistical analysis and analysed according to the group they were originally assigned, regardless of what treatment (if any) they received. This method allows the investigator to draw accurate (unbiased) conclusions regarding the effectiveness of an intervention. This method preserves the benefits of randomization, which cannot be assumed when using other methods of analysis. A mixed model ANOVA will be used to ascertain any difference across the means scores over the five data points (baseline, post-intervention, 12- and 24-week follow-up).

Hedges’ g will be used to ascertain the extent of the effect sizes from baseline, post, and three-month follow-up for the sample. Calculating an effect size is considered a useful indicator to estimate the overall effect of an intervention [24]. Overall, Hedges’ g has been found useful in estimating effect sizes in large and small samples and has also proved valuable in estimating effect sizes in clinical practice for groups that are equal or <20 [24]. The interpreting of estimated effects will be as follows: *0.2* (small effect), *0.5* (medium effect), and *0.8* (large effect).

#### Neuroimaging analysis

fNIRS statistical analysis will be performed within Satori fNIRS (NIRx, Medical Technologies, LLC, Berlin, Germany) and using JASP (Version 0.18). Specifically, hemodynamic data from the jittered block design will be analyzed using the General Linear Model (GLM) approach. Correspondingly, stimulus onset times for fearful and neutral face blocks will be convolved with inbuild canonical hemodynamic response functions in Satori fNIRS, to create regressors representing task-related neural activity (F, N, BS). The GLM will be applied separately to each participant's preprocessed HbO and deoxy signals to estimate cortical responses associated with each condition. Motion artifacts will be corrected within Satori. Group-level statistical assessments, including linear mixed effects models and *t*-tests, will be performed to identify significant activation patterns and differences between the group conditions.

#### Interview data post-intervention

Interview data will be analysed using thematic analysis (Braun & Clark, 2013) to analyse qualitative responses on dominant themes related to the feasibility and acceptability of the intervention (PE-PC) and associated neuroimaging techniques (fNIRS) used in the study.

## Limitations

The study is ongoing and has had some delays due funding, yet recruitment and training has commenced.

## Conclusion

The current study is ongoing, therefore, we do not have sufficient data to reach any empirically based conclusions. Future works will continue and expand on the integration of eurobiological markers n the use of PE-PC for PTSD in a South African setting.

## CRediT author statement

DB and SZ: Conceptualization, Methodology, Writing- Reviewing and Editing.

## Declaration of competing interest

The authors declare that they have no known competing financial interests or personal relationships that could have appeared to influence the work reported in this paper.

## Data Availability

Data will be made available once data collection is completed.

## Appendix Supplementary materials

Image, application 1

Image, application 2
