# Supplementary material for: Detecting neurobiological markers in treatment response to prolonged exposure therapy for PTSD: An RCT using functional near-infrared spectroscopy
Source: MethodsX. 2026 Mar 19;16:103867. doi: 10.1016/j.mex.2026.103867 (PMC13123371; doi:10.1016/j.mex.2026.103867)
Supplement: Supplementary file 1 [file mmc1.docx]

**Appendix A: PE-PC Overview and Key Component Review**

**Contact 0**

- Duration: 30-minute appointment
- Brief Assessment (PCL-5 (Past Month); about 10 minutes)
- Education (about 10-15 minutes)
  - Normal recovery curve; “getting stuck”
  - Role of avoidance in maintaining symptoms
  - Evidence for exposure-based treatments
- Presentation of treatment options (about 5 min)
- Primary Care vs Specialty Care vs Self-Care

**Total visit duration should be 30 minutes- Cannot MAX out all sections without going**

**too long**

**Contact 1**

- Duration: 30-minute appointment
- Patient completes PCL-5 (weekly) and PHQ-9 if possible, prior to start of the appointment.
- Present “Confronting Traumatic/Uncomfortable Memories” workbook (about 15-20 min)
  - - Review of brief rationale
    - At home: Write narrative of traumatic experience
    - At home: Answer emotional processing questions
- Taking Back from PTSD – in vivo exposure (about 5-10 min)
- Prescribe as homework (about 5 min)
  - - Goal: 30 minutes write and review daily
    - Self-monitor SUD’s
- Problem-solve homework implementation (about 5 min but often occurs during above)
  - - When/where of homework
    - Barriers to completion
- **Total visit duration should be 30 minutes- Cannot MAX out all sections without going**

**too long**

**Contact 2 to Contact 8 total**

- Duration: 30-minute appointment
- Patient completes PCL-5 (weekly) and PHQ-9 if possible, prior to start of the appointment.
- Discuss homework completion (2-5 min)
  - Review SUD’s
- Exposure (10-15 min)
  - Read narrative out loud (at least once)
  - Read answers to processing questions out loud
- Processing of the meaning of the trauma (10-15 min)
- Homework (5-10 min)
  - Re-assign writing assignment as homework
  - Encourage opportunities for in vivo exposure (Taking Back from PTSD)
- Last Contact: Review progress and relapse prevention (during processing time)

• **Total visit duration should be 30 minutes- Cannot MAX out all sections without going**

**too long**
