# Supplementary material for: Detecting neurobiological markers in treatment response to prolonged exposure therapy for PTSD: An RCT using functional near-infrared spectroscopy
Source: MethodsX. 2026 Mar 19;16:103867. doi: 10.1016/j.mex.2026.103867 (PMC13123371; doi:10.1016/j.mex.2026.103867)
Supplement: Supplementary file 2 [file mmc2.docx]

**Appendix B**

**Supportive Counselling Key areas**

Supportive Counselling, also known as person-centred counselling, is a non-directive approach to assist persons with various forms of psychological distress.

- Counseling is provided in brief sessions using these techniques:
- Active listening
- Empathy
- Unconditional positive regard
- Advice-giving
- Adding perspective
- Confirmation of the appropriateness of patient concerns

**Focus on Solutions**

Empathize with the patient while moving the dialogue towards the construction of clear, simple, specific behavioral change plans:

• Work

• Home

• Finances

• Health

**Focus on Coping Strategies**

Coping strategies can be divided into problem-focused, which are directed at situations that can be changed, and emotion-focused, which are directed at situations that cannot be changed. After helping a patient recognise whether a situation can be changed, the following helpful coping strategies may be suggested to patients.

The above-mentioned components can be implemented at various stages of sessions. As mentioned, supportive counselling is non-directive and does not prescribe what to do in sessions.
